# Do not forget the children: a model‐based analysis on the potential impact of COVID‐19‐associated interruptions in paediatric HIV prevention and care

**DOI:** 10.1002/jia2.25864

**Published:** 2022-01-20

**Authors:** Clare F. Flanagan, Nicole McCann, John Stover, Kenneth A. Freedberg, Andrea L. Ciaranello

**Affiliations:** ^1^ Medical Practice Evaluation Center Massachusetts General Hospital Boston Massachusetts USA; ^2^ Avenir Health Glastonbury Connecticut USA; ^3^ Harvard Medical School Boston Massachusetts USA; ^4^ Division of Infectious Diseases Massachusetts General Hospital Boston Massachusetts USA; ^5^ Division of General Internal Medicine Massachusetts General Hospital Boston Massachusetts USA; ^6^ Department of Health Policy and Management Harvard T.H. Chan School of Public Health Boston Massachusetts USA; ^7^ Harvard University Center for AIDS Research Cambridge Massachusetts USA

**Keywords:** COVID‐19, HIV, paediatric HIV, maternal‐to‐child transmissions, pregnant and breastfeeding women living with HIV

## Abstract

**Introduction:**

The COVID‐19 pandemic has affected women and children globally, disrupting antiretroviral therapy (ART) services and exacerbating pre‐existing barriers to care for both pregnant women and paediatric populations.

**Methods:**

We used the Spectrum modelling package and the CEPAC‐Pediatric model to project the impact of COVID‐19‐associated care disruptions on three key populations in the 21 Global Plan priority countries in sub‐Saharan Africa: (1) pregnant and breastfeeding women living with HIV and their children, (2) all children (aged 0–14 years) living with HIV (CLWH), regardless of their engagement in care and (3) CLWH who were engaged in care and on ART prior to the start of the pandemic. We projected clinical outcomes over the 12‐month period of 1 March 2020 to 1 March 2021.

**Results:**

Compared to a scenario with no care disruption, in a 3‐month lockdown with complete service disruption, followed by 3 additional months of partial (50%) service disruption, a projected 755,400 women would have received PMTCT care (a 21% decrease), 187,800 new paediatric HIV infections would have occurred (a 77% increase) and 516,800 children would have received ART (a 35% decrease). For children on ART as of March 2020, we projected 507,200 would have experienced ART failure (an 80% increase). Additionally, a projected 88,400 AIDS‐related deaths would have occurred (a 27% increase) between March 2020 and March 2021, with 51,700 of those deaths occurring among children engaged in care as of March 2020 (a 54% increase).

**Conclusions:**

While efforts will continue to curb morbidity and mortality stemming directly from COVID‐19 itself, it is critical that providers also consider the immediate and indirect harms of this pandemic, particularly among vulnerable populations. Well‐informed, timely action is critical to meet the health needs of pregnant women and children if the global community is to maintain momentum towards an AIDS‐free generation.

## 1 INTRODUCTION

While antiretroviral therapy (ART) coverage has been increasing globally, coverage among children lags behind that of adults [1], with 74% of adults receiving ART compared to only 53% of children in 2020 [2]. Even before COVID‐19, HIV programmes were falling short of global treatment targets [3]. Some regions, including West and Central Africa, experienced backsliding after marked progress over years, with ART coverage of pregnant women peaking at 61% in 2016 before declining to 57% in 2019 [4].

Although SARS‐CoV‐2 infections have largely spared paediatric populations to date [5], the secondary impact of this pandemic has affected women and children globally [6]. This pandemic, like so many crises before, has disproportionately affected women economically, socially and in terms of health [7]. Here, we consider how the pandemic has exacerbated pre‐existing barriers to care for pregnant women and paediatric populations [3].

Disruption of ART services was reported in 36 countries worldwide between April and June 2020 [8]. For some, public health measures, such as lockdowns, made it more difficult to access healthcare facilities. Even when facilities remained open, people were fearful of contracting SARS‐CoV‐2 while accessing services [9]. Concern about SARS‐CoV‐2 risk was especially relevant for pregnant women, both early in the pandemic, when data were lacking regarding how the virus would affect pregnant women and their infants [10], and later, when new data indicated correlation between COVID‐19 and adverse birth outcomes [11].

While lockdowns have been lifted, permitting travel to healthcare facilities, the pandemic has strained global supply chains, contributing to increased ART costs. These strains also put countries at risk for ART stockouts [12], with formulations used for postnatal prophylaxis and paediatric ART at particular risk, given the limited number of paediatric ART manufacturers and supply chain issues pre‐dating COVID‐19. Meanwhile, many of the major AIDS relief donors face pandemic‐related economic hardship [13].

We used the Spectrum software package and the Cost‐Effectiveness of Preventing AIDS Complications Pediatric (CEPAC‐P) model to project the potential impact of service interruptions on key outcomes for children at risk for or living with HIV.

## 2 METHODS

Spectrum is a modelling package that generates estimates of HIV‐related measures. For model inputs, Spectrum uses country‐specific surveillance, survey and programme data, such as the number of children receiving ART and cotrimoxazole and the number of women receiving antiretroviral prophylaxis by regimen. Results from scientific studies are used to inform parameters for which country‐specific estimates do not exist, such as MTCT rates by regimen and mother's CD4 count, and the effect of ART on mortality [14].

The CEPAC‐P model is a clinically detailed microsimulation model of paediatric HIV acquisition, disease progression and treatment. CEPAC‐P was initially populated, validated and calibrated using cohort data from the International epidemiology Databases to Evaluate AIDS (IeDEA) East African cohort. The model was then calibrated to match pooled UNAIDS data from eight sub‐Saharan African countries to ensure generalizability of results to the broader region [15]. These data were shared under data use agreements. This work was approved by the Mass General Brigham Institutional Review Board. No primary data were collected for this analysis. Thus, no consent processes were implemented. Model‐based analyses were conducted between September 2020 and March 2021.

Drawing on the respective strengths of these models, we simulated three separate populations likely to be affected by COVID‐19‐associated care disruptions in the 21 Global Plan priority countries in sub‐Saharan Africa [16]. Using Spectrum, we projected the impact of COVID‐19‐associated care disruptions on: (1) pregnant and breastfeeding women living with HIV and their children, and (2) all children living with HIV (CLWH) (aged 0–14 years), regardless of their engagement in care. Projected Spectrum outcomes include the number of women engaged in PMTCT care (defined as pregnant and breastfeeding women receiving effective ART), new paediatric HIV infections, CLWH receiving ART and deaths among all CLWH. Using the CEPAC‐P model, we further explored the impact of care disruptions on CLWH who were engaged in care and on ART pre‐pandemic. CEPAC‐P projected the proportion of children alive and virologically suppressed on ART. These proportions were then applied to Spectrum‐generated estimates of the number of children on ART in March 2020.

We then projected clinical outcomes from 1 March 2020 to 1 March 2021. We first modelled a comparator scenario with no service interruption. In all disruption scenarios, we modelled time‐limited disruption of ART access. Disruption duration varied between scenarios: all included 3 months of complete lockdown, with 100% of the population unable to access ART; for the subsequent 3 months of disruption, we varied the proportion of the population that remained unable to access services (25%, 50%, 75% and 100%). Following these modelled 6‐month disruptions, we assumed a full return to pre‐disruption access to care.

Of the overall paediatric cohort, 65% were modelled as virologically suppressed pre‐disruption [17]. Upon re‐initiating ART, children who had suppressed pre‐disruption had a 91% probability of re‐suppressing [18]. The 35% of children who were failing ART pre‐disruption were modelled to continue failing upon re‐initiating ART; upon detection of failure, they started second‐line ART with a 75% probability of viral suppression at 24 weeks [18, 19].

## 3 RESULTS AND DISCUSSION

If there had been no service interruption due to COVID‐19, we projected that between March 2020 and March 2021 within the 21 priority countries in sub‐Saharan Africa, 959,200 women would have received PMTCT care, 106,200 new paediatric HIV infections would have occurred and 796,700 children would have received ART (Table 1, second column). Of those children on ART as of March 2020, we projected that there would have been 282,400 ART failures. We projected 73,400 AIDS‐related deaths would have occurred between March 2020 and March 2021, with 33,700 of those deaths occurring among children engaged in care as of March 2020.

**Table 1:** Modelled results: change in projected outcomes as compared to scenario without any service disruption, from the Spectrum and CEPAC‐P models

Service disruption length		No disruption (comparator)	3 months (100%)	3 months (100%)	3 months (100%)	6 months (100%)
			3 months (25%)	3 months (50%)	3 months (75%)	
Mothers receiving PMTCT^a^ ^,^ ^b^						
Projected number		959,200	789,800	755,400	721,200	685,200
	# change	n/a	*−169,400*	*−203,800*	*−238,000*	*−274,000*
	% change		*−18%*	*−21%*	*−25%*	*−29%*
New paediatric HIV infections^a^						
Projected number		106,200	175,400	187,800	200,000	213,500
	# change	n/a	*+69,200*	*+81,600*	*+93,500*	*+107,300*
	% change		*+65%*	*+77%*	*+88%*	*+101%*
Children receiving ART^a^						
Projected number		796,700	565,300	516,800	468,000	418,500
	# change	n/a	*−231,400*	*−279,900*	*−328,600*	*−378,100*
	% change		*−29%*	*−35%*	*−41%*	*−47%*
ART failures among children engaged in care prior to service disruptions^c^ ^,^ ^d^						
Projected number		282,400	458,900	507,200	555,400	603,700
	# change	n/a	*+176,500*	*+224,800*	*+273,000*	*+321,300*
	% change		*+63%*	*+80%*	*+97%*	*+114%*
Paediatric AIDS‐related deaths among all children living with HIV^a^						
Projected number		73,400	85,900	88,400	90,900	94,000
	# change	n/a	*+28,900*	*+33,900*	*+38,700*	*+44,300*
	% change		*+23%*	*+27%*	*+30%*	*+35%*
Paediatric AIDS‐related deaths among all children engaged in care^c^						
Projected number		33,700	49,200	51,700	54,100	56,500
	# change	n/a	*+15,600*	*+18,000*	*+20,400*	*+22,900*
	% change		*+46%*	*+54%*	*+61%*	*+68%*

*Note*: Numbers may not sum due to rounding.

Abbreviations: ART, antiretroviral therapy; PMTCT, prevention of maternal‐to‐child transmission.

^a^ Model projections generated by Spectrum.

^b^ Mothers receiving PMTCT is defined as pregnant and breastfeeding women receiving effective ART.

^c^ Model projections generated by CEPAC‐P.

^d^ ART failure within the CEPAC‐P model includes (1) clinical treatment failure, defined as a new or recurring WHO Stage 3/4 opportunistic infection or tuberculosis, (2) immunologic treatment failure, defined as a CD4% <10% (for children <5 years old) or CD4 count <100/μl (for children ≥5 years old) or (3) virologic treatment failure, defined as an increase of 3 or more viral load (VL) strata following ART start, with strata copies/ml defined as 0–20; 20–500; 500–3000; 3000–10,000; 10,000–30,000; 30,000–100,000; and >100,000.

**Figure 1:**
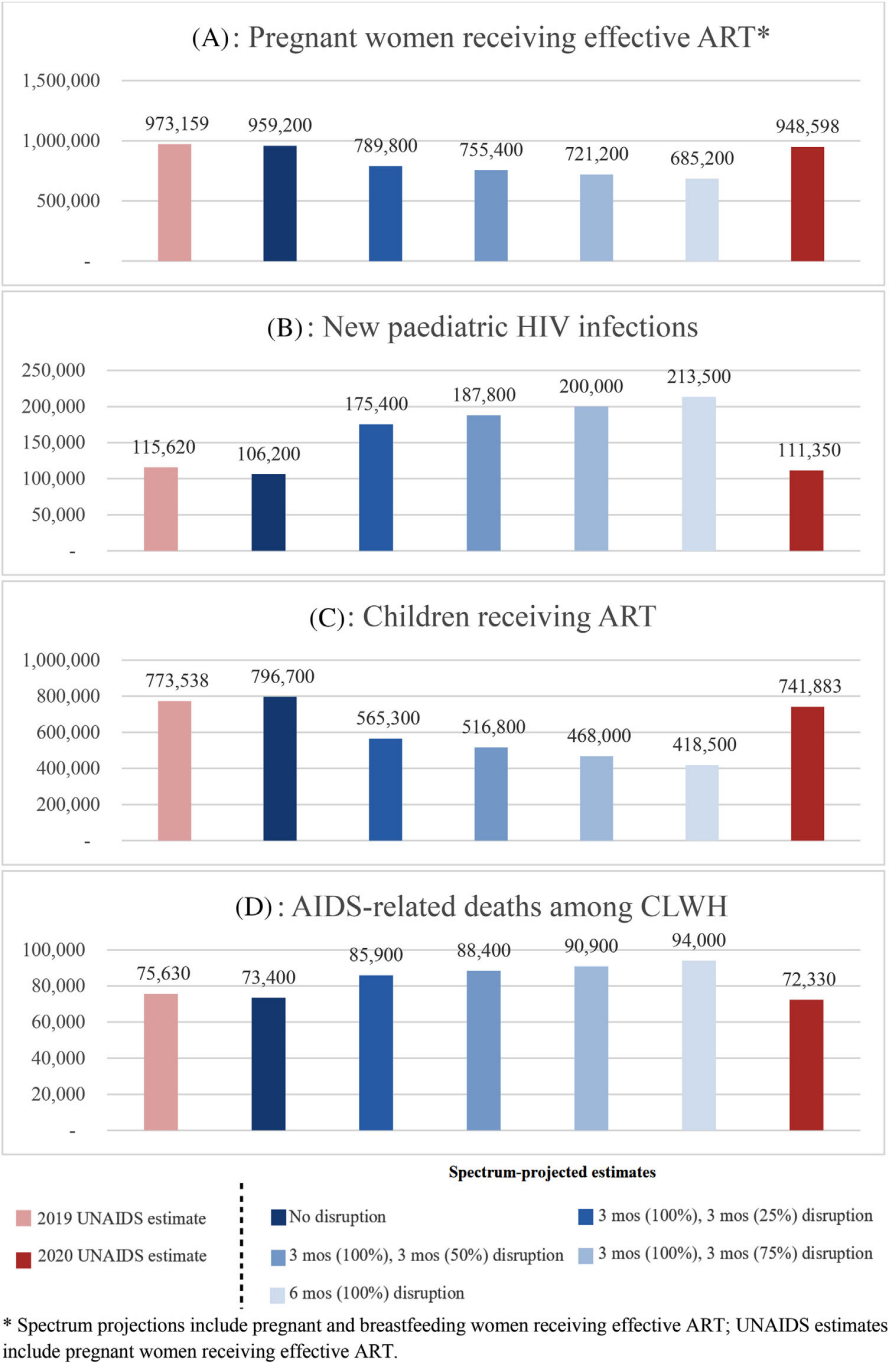
Modelled Spectrum results, alongside 2019 and 2020 UNAIDS estimates. * Spectrum projections include pregnant and breastfeeding women receiving effective ART; UNAIDS estimates include pregnant women receiving effective ART.

With a 3‐month lockdown resulting in complete service disruption, followed by an additional 3 months of partial (50%) service disruption, we projected 755,400 women would have received PMTCT care (a 21% decrease), 187,800 new paediatric HIV infections would have occurred (a 77% increase) and 516,800 children would have received ART (a 35% decrease; Table 1, fourth column). For children on ART as of March 2020, we projected 507,200 would have experienced ART failure (an 80% increase). A projected 88,400 AIDS‐related deaths would have occurred (a 27% increase) between March 2020 and March 2021, with 51,700 of those deaths occurring among children engaged in care as of March 2020 (a 54% increase). Table 1 displays results from additional simulated scenarios. Figure 1 displays Spectrum projections alongside UNAIDS estimates.

Empiric evidence on the impact of COVID‐19‐associated service interruptions on maternal and paediatric health is likely to emerge over the next several years. While awaiting these data, we brought together projections from two validated and widely used models to estimate potential outcomes for women and children affected by HIV in the 21 Global Plan priority countries in sub‐Saharan Africa. We found that 3 months of complete service interruption, followed by 3 additional months of a 50% service interruption would greatly impact paediatric HIV outcomes, with projected new HIV infections increasing by 77%, ART failures increasing by 80% and AIDS‐related deaths increasing by 27%.

These estimates are conservative for at least three reasons. First, they do not account for increases in maternal HIV incidence that may have resulted from care interruptions affecting ART access for male partners or pre‐exposure prophylaxis (PrEP) for women, or pandemic‐spurred economic hardships that may have put women at increased risk for HIV infection [20, 21], although this may have been at least partially offset in some settings by reduced sexual contact during lockdowns [22], or by increased PrEP enrolment due to COVID‐inspired programme adaptations [23]. These projections also do not account for interruptions in HIV testing services that may have impacted maternal diagnoses. Second, we exclude the possible impact of maternal illness, loss to follow‐up or death on paediatric care outcomes, such as retention, viral suppression and survival [24]. Finally, the CEPAC‐P projections excluded CLWH who were not in care pre‐pandemic, many of whom may have missed an opportunity to initiate or re‐engage in care during the service interruption.

This analysis has two important limitations. First, we provide aggregate estimates for 21 countries, which necessarily exclude key differences in HIV care coverage pre‐COVID‐19, as well as in the length and severity of pandemic‐related care interruptions. Following international guidance, we examined the impact of uncertainty in these parameters by varying the length and extent of the modelled disruption, the most influential and uncertain input [25]. Second, infant testing is a crucial piece of the PMTCT care cascade that was likely impacted by care disruptions [26]. However, we were unable to include this important outcome due to insufficient data about pre‐pandemic rates of infant testing across the 21 priority countries.

Many questions remain about the COVID‐19 pandemic, including when it will subside. It is likely that programmes providing care for people living with HIV – and other illnesses – will be affected by pandemic‐associated service disruptions for some time to come [27]. In order to prevent critical backsliding in the global progress to prevent mother‐to‐child HIV transmission, and to increase ART access for mothers and their children, it is imperative to quantify the impact of the pandemic on care access and clinical outcomes, to inform prioritization and planning that will enable HIV treatment programmes to meet the ongoing health needs of their communities, particularly among vulnerable populations. Moreover, it will be especially important to address gaps already caused by COVID‐19‐related care interruptions.

These findings suggest an increase in projected new paediatric HIV infections, with more infants born with HIV to women temporarily disengaged from PMTCT programmes. Reports on the impact of COVID‐related service disruptions show that several countries experienced declines in both rates of HIV testing in antenatal clinics and in antiretroviral prophylaxis to prevent vertical transmissions in 2020 [28]. Many of these countries have since rebounded to pre‐COVID coverage levels [29]. However, with much of the world still struggling to roll out effective vaccines, and with so much uncertainty regarding the future of the pandemic and emerging variants, it is possible that we will see similar HIV care coverage declines in the coming months. For CLWH whose care has been interrupted, local programmes will need to take swift action to restart care, identify virologic failure and switch children to second‐line ART when appropriate.

As re‐opening progresses in many countries, meeting the needs of pregnant women and CLWH should be a top priority. This will require enhanced action and thoughtful solutions. COVID‐19 has presented an opportunity to rethink traditional care delivery in favour of more innovative solutions. Differentiated service delivery (DSD) may help to minimize the risk of COVID‐19 exposure while continuing to meet the healthcare needs of pregnant women and CLWH [30, 31]. There was an increased demand for DSD during the pandemic [32], with evidence supporting its non‐inferiority compared to the standard of care [33, 34]. Dispensing multi‐month ART prescriptions, a cornerstone of the DSD framework, can enable individuals to maintain ART adherence without frequent clinic visits, as demonstrated in Tanzania and Namibia [35, 36]. Virtual follow‐up can also function to meet healthcare needs of these populations, while limiting exposure [37], exemplified in Botswana [38].

Any potential solutions – self‐testing, service bundling, ART delivery, electronic symptom monitoring and other creative approaches [30, 31] – should consider the specific needs of the population and the barriers they face to accessing care. Pregnant women and CLWH live in an array of diverse communities, and setting‐specific needs must be considered.

## 4 CONCLUSIONS

While efforts will continue to curb the rising death tolls stemming directly from COVID‐19, providers must not lose sight of the immediate and indirect harms of this pandemic, particularly among vulnerable populations. Well‐informed timely action is critical to meet the health needs of pregnant women and children if the global community is to maintain momentum towards an AIDS‐free generation.

## COMPETING INTERESTS

The authors have no competing of interests to report.

## AUTHORS’ CONTRIBUTIONS

All authors discussed the results and contributed to the final version of this short report. NM conducted the CEPAC‐P model projections. JS conducted the Spectrum model projections. CFF and ALC had the initial idea for this analysis. CFF wrote the report.

## FUNDING

Support for CEPAC‐P model development was provided by the US National Institutes of Health (NICHD R01 HD079214 to ALC). Support for the Spectrum model was provided by the Bill & Melinda Gates Foundation (OPP1191665).

## DISCLAIMER

The content is solely the responsibility of the authors and does not necessarily represent the official views of the National Institutes of Health, the Bill & Melinda Gates Foundation or the United States Government.

## Data Availability

Data used to initially populate and calibrate the CEPAC‐P model were shared under data use agreements. No primary data were collected for this analysis.
